# Beta-Eucaine Anesthesia in Proctology

**Published:** 1910-12

**Authors:** 


### ABSTRACTS

### Beta-Eucaine Anesthesia in Proctology

Dr. Samuel Goodwin Gant, in a paper read before the Medical Society of the Borough of the Bronx (*Med. Review of Reviews*) Sept., 1910), states that his experience with beta-eucaine which extends over ten years, has been universally satisfactory. He prefers this anesthetic because it can be sterilised, does not irritate, and invariably produces a more complete anesthesia and causes less toxic manifestations than cocaine, stovaine, novocaine and like agents, when used in an equal strength and quantity. When beta-eucaine is employed in 1/8 per cent. solution in ano-rectal operations, the patient rarely complains of pain, dizziness, or turns pale, or becomes excited or faints during or following the operation. This is partly due to the non-toxic effect of the drug and partly to the fact that the solution is very weak and escapes during or following operation.

The author pleads for a more frequent use of local anesthesia, especially of infiltration anesthesia, in cases where now general narcosis is being unwarrantably used.

He enumerates those ano-rectal diseases which are operable under local anesthesia and those which require general narcosis, stating that with proper proficiency in the technic of infiltration anesthesia, which he thinks all surgeons and proctologists should take pains to acquire, he does not hesitate to recommend infiltration anesthesia in preference to general narcosis in the majority of the minor and some of the more extensive operations which the modern proctologist is called upon to perform. Of all the available agents, beta-eucaine and sterile water have given the author the most uniformly gratifying results. He prefers sterile water in operations for internal hemorrhoids and other affections having a mucous covering, but favors beta-eucaine when combinations of external hemorrhoids are to be removed and when a fissure or fistula is to be operated upon.

